# Molecular clonality and antimicrobial resistance in *Salmonella enterica* serovars Enteritidis and Infantis from broilers in three Northern regions of Iran

**DOI:** 10.1186/1746-6148-9-66

**Published:** 2013-04-05

**Authors:** Maral Rahmani, Seyed Mostafa Peighambari, Christina Aaby Svendsen, Lina M Cavaco, Yvonne Agersø, Rene S Hendriksen

**Affiliations:** 1 Department of Clinical Sciences, Faculty of Veterinary Medicine, University of Tehran, Tehran, Iran; 2 WHO Collaborating Center for Antimicrobial Resistance in Food borne Pathogens and European Union Reference Laboratory for Antimicrobial Resistance, National Food Institute, Technical University of Denmark, Kgs. Lyngby, Denmark

**Keywords:** *Salmonella* infantis, *Salmonella* enteritidis, Antimicrobial resistance, MIC determination, Resistance gene, PFGE, Fluoroquinolone, Poultry, Iran

## Abstract

**Background:**

Multidrug-resistant *Salmonella* strains are frequently encountered problems worldwide with considerable increased occurrences in recent years. The aim of this study was to investigate the occurrence and frequency of antimicrobial resistance and associated resistance genes in *Salmonella* isolates from broiler farms in different regions of Iran covering a time period of four years.

**Results:**

From 2007 to 2011, 36 *Salmonella* strains were isolated from broiler farms located in three northern provinces of Iran. The isolates were serotyped, antimicrobial susceptibility tested, and characterized for antimicrobial resistance genes associated to the phenotype. Pulsed-field gel electrophoresis (PFGE) was applied for comparison of genetic relatedness.

Two serovars were detected among the isolates; *Salmonella enterica* serovar Infantis (75%) and *S.* Enteritidis (25%). Thirty-four (94%) of the isolates exhibited resistance to nalidixic acid and ciprofloxacin caused by a single mutation in the quinolone resistance-determining region (QRDR) of *gyr*A. For all strains this mutation occurred in the codon of Asp^87^ leading to a Asp^87-^Tyr, Asp^87-^Gly or Asp^87-^Asn substitutions. All *S.* Infantis (n = 27) were resistant to tetracycline, spectinomycin, streptomycin, and sulfamethoxazole and harbored the associated resistance genes; *tet*A, *dfrA14*, *aad*A1, and *sul*I together with class 1 integrons. The isolates revealed highly similar PFGE patterns indicating clonal relatedness across different geographical locations.

**Conclusion:**

The data provided fundamental information applicable when launching future control programs for broilers in Iran with the aim to conserve the effectiveness of important antimicrobials for treatment in humans.

## Background

Salmonellosis is one of the most important diseases in both humans and animals and has been described as the second most common cause of foodborne bacterial human disease worldwide [1]. It is estimated that 93.8 million cases of gastroenteritis due to *Salmonella* spp. occur annually worldwide leading to 155,000 deaths each year [2]. A significant increase in the number of *Salmonella* infections has been observed in many countries over the past decade [3]. Globally, the most prevalent serovars in humans are *Salmonella enterica* serovars Typhimurium and Enteritidis [4].

Chicken and related products are recognized as important reservoirs for *Salmonella* and vehicles for salmonellosis. Some *Salmonella* serovars such as *S.* Enteritidis, *S.* Infantis, *S.* Kentucky, and *S.* Heidelberg appear to be more prevalent in poultry than in other food animals [5].

Widespread use of antimicrobial agents in food animal production has contributed to the occurrence in animals of resistant zoonotic pathogens such as *Salmonella*. Whilst antimicrobial resistance is moderate in the predominant poultry-related serovar; *S*. Enteritidis [5,6], multidrug resistance (MDR) is frequent in other *Salmonella* serovars [6]. In poultry production, antimicrobial agents are widely used for growth promotion, prophylaxis or treatment purposes [7,8]. As a consequence, chicken and chicken meat can harbor antimicrobial resistant strains and function as a vehicle for dissemination of these to humans. Today, MDR *Salmonella* strains are frequently encountered in most of the world and the rates of MDR have increased considerably in recent years [9]. As a result, extended-spectrum cephalosporins (ESC) and fluoroquinolones (FQ) are the drugs of choice for treatment of severe *Salmonella* infections. Accordingly, limiting the occurrence of resistance to ESC and FQ is a public health priority and these drugs have been classified by the World Health Organization as critically important antimicrobials [10]. The resistance mechanisms responsible for the increase in antimicrobial resistance are mainly a result of the horizontal gene transfer via mobile genetics elements such as plasmids and integrons. The strong association between MDR *Salmonella* and the presence of integrons; especially class 1, has been well documented [11]. To embark on and enforce prevention and control measurements, it is vital to elucidate and understand the global epidemiology of antimicrobial resistance pheno- and genotypes [12]. In recent years, MDR bacterial pathogens have emerged and disseminated in Iran and have become a challenging problem for the medical and veterinary community. Unfortunately, studies revealing the frequency of antimicrobial resistance and corresponding genes in pathogens of poultry origin are sparse. Poultry is one of the predominant reservoirs for *Salmonella* in Iran and in the Iranian poultry sector production infrastructure has progressed rapidly in recent years, especially in the northern part of the country.

In this study, the objectives were to investigate the occurrence and frequency of antimicrobial resistance and associated resistance genes in *Salmonella* isolates from broilers originating from farms located in three northern provinces of Iran in the period from 2007 to 2011 with the application of minimal inhibitory concentration (MIC) determination and polymerase chain reaction (PCR). To elucidate if any genotypes were predominating among the isolates, mechanisms for FQ resistance were investigated by subsequent sequencing of PCR amplicons. Additionally, we utilized pulsed-field gel electrophoresis (PFGE) and MIC patterns to determine the clonal relatedness and to elucidate any epidemiological links between the farms and in a temporal context.

## Methods

### Bacterial isolates

A total of 36 broiler-related *Salmonella* isolates covering a four-year period, from 2007 to 2011, were included in this study. The isolates originated from 14 different broiler farms situated in the northern provinces of Mazandaran, Guilan, and Golestan in Iran. The capacity of the farms ranged from 5,000 to 18,000 broilers per house. Samples taken included fresh randomly collected feces, cloacal feces and a limited number of carcasses at 8 to 49 days old. A randomized convenience sampling was employed and collection, isolation, and identification of *Salmonella* spp. were conducted according to standard procedures [13].

### Serotyping, antimicrobial susceptibility testing, and pulsed-field gel electrophoresis

The procedures for serotyping and PFGE of the isolates included in this study have been described previously [14]. In brief, all isolates were serotyped using slide agglutination and assigned a serotype according to the Kauffmann-White scheme [15]. In addition, all isolates were genotyped by PFGE using *Xba*I according to the CDC PulseNet protocol [16].

MIC determination was performed using a commercially prepared, dehydrated panel, Sensititre, from TREK Diagnostic Systems Ltd. The antimicrobials used and interpretative criteria applied were as follows: ampicillin, AMP (R > 8 mg/L); amoxicillin + clavulanic acid, AUG (R ≥ 32 mg/L); apramycin, APR (R > 32 mg/L); cefotaxime, FOT (R > 0.5 mg/L); ceftiofur, XNL (R > 2 mg/L); chloramphenicol, CHL (R > 16 mg/L); ciprofloxacin, CIP (R-low level: 0.064 to 1 mg/L, and R-high level > 1 mg/L); colistin COL (R > 2 mg/L); florfenicol, FFN (R > 16 mg/L); gentamicin, GEN (R > 2 mg/L); nalidixic acid, NAL (R > 16 mg/L); neomycin, NEO (R > 4 mg/L); spectinomycin, SPE (R > 64 mg/L); streptomycin, STR (R > 16 mg/L); sulfamethoxazole, SMX (R > 256 mg/L); tetracycline, TET (R > 8 mg/L); and trimethoprim, TMP (R > 2 mg/L). For interpretation of antimicrobial susceptibility test results epidemiological cut-off values according to EUCAST recommendations [17] were applied for all antimicrobials except APR, AUG, SMX, and SPE for which EUCAST values are not available to date. For AUG, SMX and SPE clinical breakpoints according to the Clinical and Laboratory Standards Institute (CLSI) were applied [18-20] and for APR the interpretation was based on research results from DTU Food due to the lack of a CLSI clinical breakpoint. Reference strain *E. coli* ATCC 25922 was used as a Quality Control according to CLSI standards [18-20].

### Screening for resistance genes and integrons

All isolates exhibiting phenotypic resistance to either of the tested antimicrobials were characterized for the presence of associated antimicrobial resistance genes. Isolates resistant to sulfamethoxazole (all *S.* Infantis) were screened for class 1 and 2 integrons using a PCR assay with specific primers. The PCR reactions were performed according to previously described conditions [21]. In brief, three different PCR reactions were performed utilizing the following primers: qacEΔ1-F/qacEΔ1-B targeting qacEΔ1, SulIB/qacEΔ1-F targeting the *sul*I and qacEΔ1 region, and *Int*I1 variable-B targeting the variable region of class 1 integrons. For class 2 integrons, two PCR reactions were used targeting the conserved and the variable region, respectively. Target genes, sequences, annealing temperature, amplicon sizes, and references of all PCR tests are listed in Table 1.

**Table 1:** Primer sequences used for the amplification of the various resistance genes

**Target genes**	**Sequence (5' to 3')**	**Annealing temp**	**Size (bp)**	**Reference**
*tet*A	5'-GTAATTCTGAGCACTGTCGC-3'	57	950	[22]
	5'-CTGCCTGGACAACATTGCTT-3'			
*tet*B	5'-CTCAGTATTCCAAGCCTTTG-3'	52	430	[23]
	5'-ACTCCCCTGAGCTTGAGGGG-3'			
*tet*C	5'-GGTTGAAGGCTCTCAAGGGC-3'	62	505	[23]
	5'-CCTCTTGCGGGATATCGTCC-3'			
*tet*D	5'-CATCCATCCGGAAGTGATAGC-3'	57	435	[24]
	5'-GGATATCTCACCGCATCTGC-3'			
*tet*G	5'-GCAGCGAAAGCGTATTTGCG-3'	62	680	[25]
	5'-TCCGAAAGCTGTCCAAGCAT-3'			
*aad*A	5'-ATTTGCTGGTTACGGTGACC-3'	56	533	[21]
	5'-CTTCAAGTATGACGGGCTGA-3'			
*str*A	5'-CCAATCGCAGATAGAAGGC-3'	55	500	[26]
	5'-CTTGGTGATAACGGCAATTC-3'			
*str*B	5'-GGATCGTAGAACATATTGGC-3'	56	500	[26]
	5'-ATCGTCAAGGGATTGAAACC-3'			
*dfrA14*	5'-TGAGAACCTTGAAAGTATCATTG-3'	55	483	This study
	5'-ACCCTTTTTCCAAATTTGATAG-3'			
*flor*R	5'-ATGGCAGGCGATATTCATTA-3'	55	320	This study
	5'-AAACGGGTTGTCACGATCAT-3'			
*sul*II	5'-GCGCTCAAGGCAGATGGCATT-3'	70	284	[26]
	5'-GCGTTTGATACCGGCACCCGT-3'			
*sul1/qacEΔ1I*	5'-ATCGCAATAGTTGGCGAAGT-3'	58	798	[22]
	5'-GCAAGGCGGAAACCCGCGCC-3			
*sul*I	5'-TGAGATCAGACGTATTGCGC-3'	58	420	This study
	5'-TTGAAGGTTCGACAGCACGT-3'			
*qacEΔ1*	5'-ATCGCAATAGTTGGCGAAGT-3'	57	226	[22]
	5'-CAAGCTTTTGCCCATGAAGC--3'			
*Int*I1	5'-AAGCAGACTTGACCTGAT-3'	55	Variable	[22]
	5'-GGCATCCAAGCAGCAAGC-3'			
*Int*I2	5'-CACGGATATGCGACAAAAAGGT-3'	60	789	[27]
	5'-GTAGCAAACGAGTGACGAAATG-3'			
*Int*I2 variable	5'-GACGGCATGCACGATTTGTA-3'	56	2214	[28]
	5'-GATGCCATCGCAAGTACGAG-3'			
*gyr*A	5'-TACCGTCATAGTTATCCACGA-3'	60	312	[29]
	5'-GTACTTTACGCCATGAACGT-3'			
*par*C	5'-CTATGCGATGTCAGAGCTGG-3'	59	261	[29]
	5'-TAACAGCAGCTCGGCGTATT-3'			

All isolates resistant to nalidixic acid and ciprofloxacin (all isolates included in this study with the exception of two *S.* Enteritidis) were tested by PCR amplification to detect possible chromosomal mutations in DNA gyrase (*gyr*A) and topoisomerase IV (*par*C). Amplicons of the genes *gyr*A and *par*C were purified using the GFX™ PCR DNA kit (Amersham Biosciences, Piscataway, New Jersey, US) and one sample of each serotype per farm was shipped to Macrogen Inc., South Korea for sequencing using the same forward primers as used in the PCR analysis. Vector NTI suite 11 (InforMax, Inc.) software (Bethesda, Maryland, US) was used for sequencing analysis and alignment. The resulting nucleotide sequences were compared to the corresponding sequences obtained from GenBank [30].

## Results

### Serotyping

Twenty-seven (75%) out of 36 isolates were typed as *S.* Infantis whereas the remaining isolates were *S.* Enteritidis (25%, n = 9). The distribution of *S.* Infantis in each province was 90%, 82%, and 50% in Mazandaran, Golestan, and Guilan, respectively, whereas the distribution of *S.* Enteritidis was 50%, 18%, and 10% in Guilan, Golestan, and Mazandaran, respectively.

### Antimicrobial susceptibility testing

**Figure 1:**
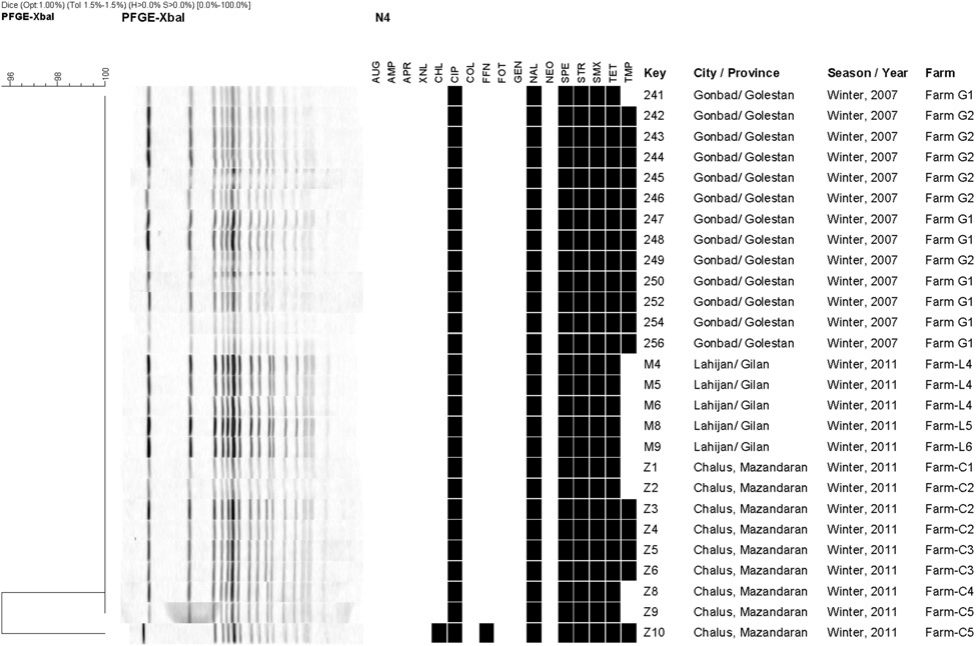
**PFGE pattern of *Salmonella *serovar Infantis isolated from three Northern provinces of Iran.** Footnotes: Black squares represent the isolates classified as resistant, Abbreviations: AMP, Ampicillin; AUG, amoxicillin + clavulanic acid; APR, apramycin; FOT, cefotaxime; XNL, ceftiofur; CHL, chloramphenicol; CIP, ciprofloxacin; COL, colistin; FFN, florfenicol; GEN, gentamicin; NAL, nalidixic acid; NEO, neomycin; SPE, spectinomycin; STR, streptomycin; SMX, sulfamethoxazole; TET, tetracycline; TMP, trimethoprim.

**Figure 2:**
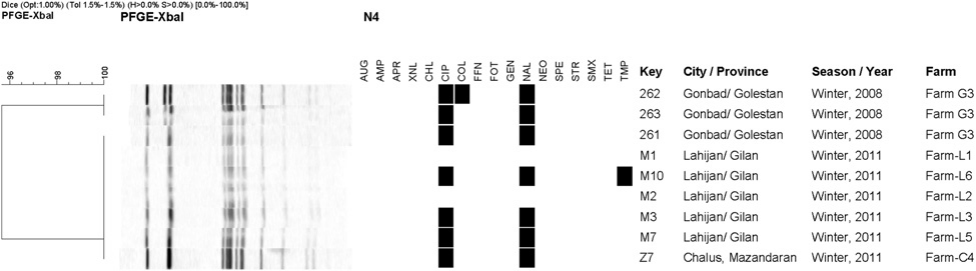
**PFGE pattern of *Salmonella *serovar Enteritidis isolated from three Northern provinces of Iran.** Footnotes: Black squares represent the isolates classified as resistant, Abbreviations: AMP, Ampicillin; AUG, amoxicillin + clavulanic acid; APR, apramycin; FOT, cefotaxime; XNL, ceftiofur; CHL, chloramphenicol; CIP, ciprofloxacin; COL, colistin; FFN, florfenicol; GEN, gentamicin; NAL, nalidixic acid; NEO, neomycin; SPE, spectinomycin; STR, streptomycin; SMX, sulfamethoxazole; TET, tetracycline; TMP, trimethoprim.

Thirty-four out of 36 isolates tested were resistant to at least two antimicrobial agents out of 17 agents tested (Figures 1 and 2). Two (5.5%) *S.* Enteritidis isolates from the same province were susceptible to all tested antimicrobial agents (Figure 2). The highest frequency (94%, n = 34) of antimicrobial resistance was observed to nalidixic acid and ciprofloxacin (Figures 1 and 2). Of the serovars identified in this study, *S.* Enteritidis showed less resistance to antimicrobial agents compared to *S*. Infantis. Of 27 *S.* Infantis isolates, all were resistant to ciprofloxacin, nalidixic acid, tetracycline, spectinomycin, streptomycin, sulfamethoxazole. In addition, 17 (63.0%) were resistant to trimethoprim, and one (3.7%) was resistant to florfenicol and chloramphenicol (Figure 1).

### Screening for resistance genes and integrons

Out of twenty-seven *S.* Infantis isolates from three provinces, all (100%) harbored the *int*l genes confirming the presence of class 1 integrons but none of the *S.* Enteritidis were positive for integron class 1. The *S.* Infantis isolates were resistant to six or more antimicrobial agents (Figure 1). Seventeen (62.9%) of the *S.* Infantis isolates from the provinces of Golestan and Mazandaran carrying the class 1 integron also harbored the class 2 integron, however, the PCR result for the detection of the variable region in integron class 2 was negative. We confirmed the presence of *Int*I in two isolates (#248 and #Z6) by nucleotide sequence comparison with that of an *Escherichia coli* strain (GenBank accession no. AU780012) from position 1038 to 1785 and found 100% similarity. Among the genes conferring resistance to tetracycline, 27 of *S.* Infantis isolates originating from all three provinces carried the *tet*A gene. However, none of *S.* Infantis isolates contained *tet*B, *tet*C, *tet*D, and *tet*G genes. In addition, no *S.* Infantis isolates were positive for the presence of *sul*II. *S.* Infantis isolates that were tested for the presence of *str*A, *str*B and *aad*A1 genes, were positive for *aadA*1 only. In addition, one *S.* Infantis isolate (2.7%) from Mazandaran province was found positive for *floR*. Twenty-two (61.1%) *S.* Infantis isolates from the provinces of Golestan and Mazandaran harbored the *dfrA14* conferring resistance to trimethoprim whereas only one (2.7%) *S.* Enteritidis isolate was positive for *dfrA14*. The detailed data of all isolates and PCR results for detected genes are shown in Table 2.

**Table 2:** **Distribution of antimicrobial resistance genes of *Salmonella *isolates originating from poultry in three Northern provinces of Iran**

**Province**	**No. of isolates**	**Serotype**	**No. and (%) of isolates with antimicrobial resistance genes**						
			*flo*R	*tet*A	*aadA*1	*dfrA14*	*sul*I	*Int*1	*Int*2
Golestan	13	*S*. Infantis	0	13 (81)	13 (81)	12 (75)	13 (81)	13 (81)	12 (75)
	3	*S*. Enteritidis	0	0	0	0	0	0	0
Mazandaran	9	*S*. Infantis	1 (10)	9 (90)	9 (90)	5 (50)	9 (90)	9 (90)	5 (50)
	1	*S*. Enteritidis	0	0	0	0	0	0	0
Guilan	5	*S*. Infantis	0	5 (50)	5 (50)	0	5 (50)	5 (50)	0
	5	*S*. Enteritidis	0	0	0	1 (10)	0	0	0
Total	36		1 (3)	27 (75)	27 (75)	18 (50)	27 (75)	27 (75)	17 (47)

All isolates were negative for detection of *tet*B, *tet*C, *tet*G, *str*A, *str*B and *sul*II resistance genes.

Twenty-one (61.7%) out of 34 isolates that were resistant to nalidixic acid and ciprofloxacin were selected for sequencing and detection of point mutations in the Quinolone Resistance Determining Region (QRDR) of *gyr*A and *par*C genes. All of the detected single mutations occurred in codon Asp87 of the *gyr*A gene. Substitution of Asp-Tyr, Asp-Gly, and Asp-Asn were observed in 13 *S.* Infantis (61.9%), one *S*. Enteritidis (4.8%), and seven *S.* Infantis (33.3%) isolates, respectively. No double mutations were found in the *gyr*A gene of the tested isolates and no mutations were identified in the *par*C gene of any of the isolates.

### Pulsed-field gel electrophoresis

#### *Salmonella* Infantis

Two distinct PFGE patterns were observed among the 27 *S.* Infantis isolates (Figure 1). All but one isolate clustered within a unique pattern covering all three provinces and the full extent of the tested time period. Sixteen of the indistinguishable isolates within the unique cluster were from the provinces of Golestan and Mazandaran and isolated in 2007 and 2011. Additionally, all of those isolates conferred resistance to the same seven antimicrobials including ciprofloxacin and nalidixic acid (Figure 1). All but one of the remaining ten identical isolates of the unique cluster were isolated in 2011, whereas one isolate was from 2007. The ten isolates were from the province of Golestan (n = 1), Mazandaran (n = 4), and Guilan (n = 5) and all shared the same antimicrobial resistance pattern of six antimicrobials. One isolate; #Z10 from the province of Mazandaran, demonstrated a unique PFGE and resistance profile compared to the other 26 isolates included this study (Figure 1).

#### *Salmonella* enteritidis

Two clusters were defined for the nine *S.* Enteritidis covering all three provinces and the full extent of the tested time period (Figure 2). One unique pattern comprised seven isolates from all three provinces whereas the second pattern only included two strains from the province of Golestan isolated in 2008 (Figure 2). All nine isolates represented resistance profiles of resistance to ciprofloxacin and nalidixic acid and two isolates additionally conferred resistance to colistin (#262, Golestan) and trimethoprim (#M10, Mazandaran) (Figure 2).

## Discussion

Currently, increasing bacterial resistance to antimicrobial agents including quinolones and FQ poses a serious problem throughout the world. To date, several reports from Iran have described the presence of resistance to antimicrobial agents such as FQ and third-generation cephalosporins that are critically important for treatment of infections in humans [31,32].

The widespread overuse and misuse of antimicrobial agents are associated with the development of resistance to these drugs that has emerged as a major problem worldwide. In Iran, patients referred to hospitals with *Salmonella* infections are usually treated with ciprofloxacin, co-amoxiclav (amoxicillin + clavulanic acid) or cephalosporins [31]. Quinolones and especially FQ are widely used in poultry farms in Iran [33], for example, enrofloxacin, which is chemically closely related to norfloxacin and ciprofloxacin, is one of the antimicrobials most frequently used for the treatment of poultry. Except for a few antimicrobials such as bacitracin and virginamycin, the use of antimicrobials for growth promotion is illegal in Iran, however, one could speculate if growth promotion in some cases might still take place. This might explain the high frequency of resistance to FQ in this study. Unfortunately, there is no surveillance or control system to monitor the prudent use of antimicrobials in the agricultural sector.

A high frequency of resistance to ciprofloxacin and nalidixic acid in *S.* Enteritidis and *S*. Infantis isolated from different regions over time within Iran was found in this study. This correlates with several studies performed in Iran where Morshed and Peighambari [34] observed high levels of resistance to nalidixic acid (24.1%) and enrofloxacin (6.9%) among *Salmonella* isolated from poultry. In comparison, they found lower frequencies of resistance to other antimicrobial drug classes. Rad et al. [33] reported 40% and 23% resistance to nalidixic acid and enrofloxacin, respectively, in *Salmonella* isolates of animal origin. The high resistance rate (94%) to quinolones observed in this study might indicate common use of enrofloxacin for various reasons during the past decade in veterinary medicine in Iran.

As in the present study, resistance to streptomycin, spectinomycin, tetracycline, trimethoprim, and sulfamethoxazole have also frequently been reported in other studies on poultry products [34-36]. The very low rate of resistance to chloramphenicol among the studied isolates (one *S.* Infantis) may be attributed to its banned use in animal production due to the potential hazards to human consumers’ health. In addition, we observed no cephalosporin resistance among the studied isolates which is valuable to the community as cephalosporin resistance is a serious public health concern.

Increased MDR has been reported in *Salmonella* isolates in many countries including Iran [31,37]. The high level of MDR observed among *S*. Infantis is in agreement with several studies from different countries which have identified healthy poultry as a potential reservoir of *S.* Infantis [35,36,38].

Furthermore, several studies documented the presence of large plasmids in *S*. Infantis carrying antimicrobial resistance (AMR) determinants. Kehrenberg et al. [39] reported transferable plasmid mediated fluoroquinolone resistance (*qnr*S1) in *S*. Infantis isolates from broilers in Germany. Gal-More et al. and Nogrady et al. [40,41] showed multidrug resistant emerging clones characterized by a large conjugative plasmid harboring the Tn*1721* transposon, including the *tetA* gene.

In contrast, *S.* Enteritidis was less prone to acquiring resistances than other serotypes as described previously in Spain and other countries [6].

In this study, we describe the presence of class 1 and 2 integrons only attributed to *S*. Infantis. Isolates containing class 1 integrons all conferred resistance to sulfonamides and harbored the *sul*I resistance gene. Additionally, all antimicrobial resistance phenotypes harbored the following associated resistance genes: streptomycin (*aadA1*), trimethoprim (*dfrA14*), florfenicol (*flo*R), and tetracyclines (*tet*A). The relationship between the application of quinolones and the dissemination of bacterial resistance from animals to humans has been described by several studies [42,43]. The results obtained in the current study could be related to those obtained by Tajbakhsh et al. [31] who described Iranian patients infected with *S.* Infantis which were resistant to the same antimicrobial agents and harbored the same resistance determinants as described in this study [31].

Presently, we are in a transition phase where next generation sequencing (NGS) technology is introduced for prediction of resistance phenotypes. Hence, linking resistance genes with resistance phenotypes will be more and more important for the understanding of why some isolates are extremely resistant and why some contain multiple resistance genes encoding resistance to the same antimicrobial classes. Recently, a study described extremely resistant *Salmonella* Senftenberg isolates harboring an arsenal of resistance genes detected by NGS and the use of a resistance database [44,45]. Many questions are still left unanswered, for example how much each of the genes affects the overall MIC value for the specific antimicrobial class.

Seventeen of the *S.* Infantis isolates also harbored class 2 integrons. For those isolates, the primers utilized for the variable region did not amplify any DNA fragment. This is probably due to not previously observed variations or large insertion in the variable region. Overall, the presence of integrons indicates the important role of those in disseminating resistance determinants in *S.* Infantis. Additionally, this indicates a possible spread of the resistant *S.* Infantis isolates from poultry to humans. Unfortunately, PFGE was not applied in the study of Tajbakhsh et al. why a comparison of PFGE profiles could not be conducted to determine the genetic relatedness.

The serovars of this study predominantly belonged to *S.* Infantis followed by *S*. Enteritidis. EFSA and ECDC [46] have considered *S.* Infantis as the third most common serovar in European patients since 2006 with an increase from 1.0% to 1.6% in 2009 in the European Union (EU). Additionally, the same trend has been observed globally, where the overall proportion of *S.* Infantis over the years 2001 to 2007 increased from 1.5% to 2.2% [4]. Likewise, chicken meat has been acknowledged as a significant source of *S.* Infantis transmitting the infection to humans [47]. To the best of our knowledge; this is the first study that links *S.* Infantis to poultry in Iran. However, there are several reports in human medicine indicating that *S.* Infantis is an important contributor to human salmonellosis. Hamidian et al. [32] found 20.9% *S.* Enteritidis and 5.4% *S.* Infantis from 129 *Salmonella* isolated from humans in Tehran. In addition, the highest level of resistance was observed to nalidixic acid whereas no resistance to ciprofloxacin was observed. The absence of resistance to ciprofloxacin in previous reports most likely reflects the interpretative criteria applied.

All sequenced isolates resistant to nalidixic acid and ciprofloxacin presented a single-mutation in Quinolone Resistance-Determining Regions of *gyr*A in codon 87. The percentage of strains with mutation in codon 87 is consistent with studies done by San Martin et al. and Liebana et al. [48,49].

No mutation was detected in *par*C-genes. The high prevalence of *S*. Infantis in this study may suggest an outcome of a clonal expansion and establishment of specific PFGE biotypes of *S*. Infantis. Possibly, *S.* Infantis has developed mechanisms protecting the serovar from major genetic rearrangements or horizontal genetic transfers. Another explanation could be that the serovar has a recent ancestor and therefore broad dissemination has not yet been possible of the limited number of accumulated major evolutionary changes at this point of time [35].

Based on PFGE*,* indistinguishable patterns were observed among *S.* Enteritidis. The lack of genetic diversity observed for *S.* Enteritidis as determined by PFGE is also shown in previous studies that confirm this serotype as highly clonal [50]. PFGE was of limited value in the epidemiological analysis of these particular isolates, however, this may actually be a reflection of the restricted clonal diversity of pathogenic strains of *S.* Enteritidis [51]. Lack of inclusive studies about serotypes distribution, antimicrobial resistance pattern and molecular investigation of poultry isolates in Iran, make this study unable to obtain confirmatory comparisons of the results. The limitations of this study include a confined geographical coverage and limited number of isolates as well as an overall comparison approach that did not include an in-depth molecular characterization of plasmids etc. The combined effect of these is reflected in the research output and it is therefore suggested that future research should take these limitation into consideration when characterizing *S.* Infantis in both humans and poultry.

## Conclusion

The study revealed a high frequency of resistant *S.* Infantis in broilers farmed in three different geographical areas between 2007 and 2011. The isolates especially exhibited resistance to nalidixic acid and ciprofloxacin caused by a point mutation in QRDR of *gyr*A. Additionally, the isolates harbored class 1 and 2 integrons and contained the *aad*A1, *sul*I, *tet*A, and *dfrA14* resistance genes. This may suggest widespread misuse or overuse of antimicrobial agents by poultry farmers in Iran. However, the results obtained from serotyping and PFGE patterns are practical for determining the current distribution of MDR serovars of *Salmonella* and epidemiological state of *Salmonella* isolates circulating among poultry. This study showed that guidelines are needed in Iran for empiric antibacterial therapy based on a local experience of antimicrobial susceptibility testing and restriction of antimicrobial growth promoters and other drugs used without prescription in animals. In addition, the data presented in this study provided valuable fundamental information for future national control programs in Iran to conserve the effectiveness of medically important antimicrobials for treating diseases in humans.

## Competing interest

None of the authors have any conflict of interests.

## Authors’ contributions

MR and SMP provided epidemiological data and isolates. SMP, YA and LMC helped to draft the manuscript. MR and CAS carried out the MIC determination, identified antimicrobials resistance genes, and conducted the pulsed field gel electrophoresis. YA and LMC assisted detecting and identifying the integron and fluoroquinolone part of the study. RSH conceived of the study, participated in design, the analysis, and helped to draft the manuscript. MR participated in the design, coordination, conducted the analysis and drafted the manuscript. All authors read and approved the final manuscript.
